# Foreign Correspondence

**Published:** 1843-09-30

**Authors:** 


### FOREIGN CORRESPONDENCE.

PARIS, August 10, 1843.

*The Academy of Sciences—Dr. Donné's Investigations of Milk—MM. Danger and Flandin's Researches on Poisoning by Copper—Pellagra—The Microscope and Test-tube—Hematology taking the place of Medicine—Mandl's Researches on the formation of the Tartar of the Teeth.*

To the Editor of the Medical Examiner.

SIR,—Several of the recent meetings at the Academies of Science and of Medicine have been highly interesting.

At the session of Monday, the 24th ult., Dr. Donné read an interesting essay on milk, considered in relation to domestic economy and public hygiene. It appears that the adulterations of this article are becoming more grave and serious, and when the immense quantity daily consumed is considered, the importance of these investigations will be readily conceived. The milk furnished to the various hospitals of the metropolis is particularly vile. In place of yielding eight or ten per cent. of cream, as ordinary milk, it hardly gives three or four; it is besides very much diluted with water, and has generally been boiled. This latter precaution, in the actual state of science, seems necessary, to preserve it during hot weather, but it has also the inconvenience of rendering it indigestible. A milk-vender, talking to Dr. D., on the subject, and adverting to the present dearness of forage and consequent rise of milk in price, said,—« We ordinarily add water to our milk, but this year we add a little milk to our water." One cause of this sophistication is the low price paid to the hospital-furnishers, only nineteen centimes, (about three cents) a litre, (about a quart.) Another reason Dr. D. thinks, is the absence of any easy, quick, and exact means to detect the adulteration. Dr. D. has invented an instrument, the lastoscope, which, he thinks, meets the indication. He then enters into the consideration of various methods to ameliorate this state of things, and render this necessary commodity of medium purity. It is said that not less than four to five thousand litres of pure milk are daily brought to Paris; this is distributed to retail venders, and in their hands undergoes various modes of treatment. Most of the milk sold in Paris comes from a radius of from ten to fifteen leagues. The farmers are paid fifteen centimes the litre, by the wholesale merchants, who sell it again to the dealers for twenty or twenty-five centimes, who retail it to the consumers for twenty to twenty-five centimes the litre, according to its quality, the quantity of water added, and the quarter in which it is sold. Now, if milk could be transported like many other substances from any distance, without injury, competition would be greater, the price less, and a purer article furnished. The surest, simplest, and cheapest method to effect this is by ice; such is the efficacy of this means, that milk is preserved, without any trace of change, for more than fifteen days, whatever the character of the external temperature, the variations of the atmosphere, or its electric condition. The apparatus of tin, covered with wood, consists of two concentric cylinders, the interior one holding ice, the exterior one, twice the size, eontains the milk; both communicate exteriorly by openings and cocks. The value of those hospital observations based on a milk diet will be readily appreciated. The attention of the proper authorities will no doubt be called to diminish the fraud, and ameliorate the conditions of sale.

MM. Danger and Flandin are pursuing with ardor their toxicological researches. They have recently read at the Academy a very valuable memoir on poisoning by Copper. These young men, full of enthusiasm and independent in their position, have done, and will yet do much for the subject of mineral poisons. The present report has excited as much attention in the scientific world, as the famous one on arsenic, and was listened to with great attention and interest by the Academy, and was referred to the same committee, which examined their former researches. Among other matters of importance they have discovered a new symptom of poisoning by copper, which heretofore has been entirely overlooked by toxicologists. This is salivation, or rather a copious bronchial secretion, which exhibits itself ordinarily a few hours after poisoning, and in which the poison is found; thus revealing the means by which the economy frees itself from the noxious elements in the same manner as the kidneys eliminate arsenic and antimony. For nine months MM. Danger aud Flandin daily poisoned a dog with copper, and were never able to detect any evidence of the metal in the urine, the organs of the renal secretion appearing impenetrable to that metal. No traces of the poison were found in the bones or viscera. Their investigations have induced them to deny the presence of copper in a natural state in the human body. This result was obtained by direct analyses, much more delicate than those heretofore employed, as well as by physiological experiments analogous to those by which the same observers proved that there is not only no arsenic in the human organism, but that all poisonous substances are incompatible with a healthy condition of the organs. These experiments were to mix, during nine months with the food given to the dogs, sometimes the sulphate, sometimes the acetate of copper. The dose, each day accurately weighed, was gradually increased. They were enabled to take, without any effect on their health, ten centigrammes of copper in the twenty-four hours, given at their meals, so that the quantity taken of it in 263 days was not less than twenty-five grammes. During life no trace of the metal was detected in the urine, and after death none was found either in the bones, muscles, or viscera, although they were examined with the greatest care.

The success of cod liver oil in the treatment of the strumous affections of children, more particularly those of the osseous system, induced Dr. Péreyra of Bordeaux to try its effects in phthisis pulmonalis, and, as he affirmed with considerable success. Dr. Trousseau has recently experimented with this treatment at the Neckar Hospital. The subjects selected were well-marked cases of the disease—there were bloody sputa, dulness of the summits of one of the lungs, gurgling respiration, with emaciation, night sweats, fever, and all the symptoms in fact, which make a hopeless prognostic. A considerable amelioration followed the use of the medicine;—to use the words of Dr. T., they coughed and spat less, and there was less fever, and fewer night sweats, but whether this was due to the season of the year (summer) or to the remedy, time and future experiments can alone determine.

Nothing can now be done in the way of diagnosis without the test tube and the microscope. The scalpel, physical exploration, the pulse, &c., all are forgotten; reagents, little furnaces, glass tubes, have taken their place. Enter an hospital and the first thing which strikes the eye is a little table, on which all the instruments of analytical medicine are placed. The pulse is touched, perhaps the lungs examined, all, however, for form's sake; and then the serious business of the day commences. An experimental bleeding (une saignée exploratrice) is made, to begin the investigations; after it has set, it is weighed, measured, calculated, formularised, and atomised, and then by a hundred thousandth of a milligramme they are enabled to distinguish the blood of phthisis from that of rheumatism. Blood is necessary to a patient, in order that you may be enabled to tell what is the matter with him. Some of the older practitioners, who are impenetrable to the advances of science, affect to be sometimes shocked at seeing an anemic patient, or one in the last stage of phthisis, daily losing the blood which they, in their ignorance, were in the habit of cherishing. But science now consists not in curing a patient, but in discovering the exact modifications his sanguine fluid has undergone.

That fearful malady, *pellagra*, so well known in Venitian Lombady, is exciting much attention here at present. The first case occurred some months since in the service of Dr. Gibert, at St. Louis, and was recognised by M. Théophile Roussel, one of the interns, who, during a residence in Italy, was enabled to observe it. Dr. Devergie presented a case to the Academy on the 25th ult., which was of some standing, but which had not been recognized by either Dr. Biett, or later by Dr. Cazenave. Another case is said to be in the wards of Dr. Gibert. This interesting disease is probably more common than is generally thought, and like glanders, since the attention of the profession has been called to it, will now be frequently met with. It seems that for some years past, [since 1818, and was then first observed in the Canton of La Teste—Ed. M. E.] it has prevailed in portions of Gascony. Dr. Leon Marchand read, on the 25th of July, a condensed memoir of intense interest on this disease at the Academy of Medicine. Since 1836 this physician has been actively engaged in the study of the affection. He has found it to prevail in low marshy districts, where the air is dry, the soil not sterile, and water scanty and bad. In the locations where pellagra is met with, vegetation is feeble, and the domestic animals are extremely thin and small. The general symptoms are an erythematous condition of the skin of the back of the hand, of the sides of the neck, and sometimes the face. General debility occurs sooner or later, with enfeebled nervous power, tending to idiocy. The disease declines on the approach of cold weather to re-appear in the spring. The first case, that of Dr. Gibert, died. Dr. Marchand had no opportunity to make any autopsies, though death was not unfrequent. It is hereditary; he has seen five generations pellagrous.

At the session of the Academy of Sciences on the first instant, M. Mandl, the microscopist, read an interesting memoir on the composition of the tartar encrusting the teeth, and the mucous of the mouth. The result of his observations is, that the tartar of the teeth is not a deposit of calcareous matter from the saliva, or a peculiar secretion, but a mass of calcareous skeletons of infusoria, agglutinated by dry mucus, in the same manner as some soils are composed, according to the researches of Ehrenberg, almost entirely of fossil infusoria. M. M. states that if a little mucus taken from between the teeth, is placed in a drop of warmed distilled water, by the aid of a power of 400 or 500 a crowd of infusoria will move about in all directions. Their form is identical with the form described by authors as *vibrios*, and have great analogy with the *vibrios-bagnettes.* Leeuenhoëck first saw these animals in the secretions from mucous surfaces, and described them These infusoria were particularly observed in the mucus taken from the mouths of persons fasting, and constitute also a large part of the mucous coatings of the tongues of dyspeptics. If a portion of concrete tartar of the teeth be placed in a drop of water for twenty or thirty minutes, and put between two glasses, it will be seen that the tartar is composed of dead infusoria; the quantity varies very much. These infusoria are provided with a calcareous covering, since tartar, which is composed chemically of calcareous salts, is made up principally of vibrios.

